# Low salinity stress increases the risk of *Vibrio parahaemolyticus* infection and gut microbiota dysbiosis in Pacific white shrimp

**DOI:** 10.1186/s12866-024-03407-0

**Published:** 2024-07-25

**Authors:** Yi-Ting Chang, Wan-Ting Huang, Ping-Lun Wu, Ramya Kumar, Han-Ching Wang, Hsiao-Pei Lu

**Affiliations:** 1 Department of Biotechnology and Bioindustry Sciences, College of Biosciences and Biotechnology, National Cheng Kung University, Tainan, Taiwan; 2 International Center for Scientific Development of Shrimp Aquaculture, National Cheng Kung University, Tainan, Taiwan

**Keywords:** Aquaculture disease risk, Low salinity stress, Gut microbiota, Microbiota resilience, Shrimp farming

## Abstract

**Background:**

Extreme precipitation events often cause sudden drops in salinity, leading to disease outbreaks in shrimp aquaculture. Evidence suggests that environmental stress increases animal host susceptibility to pathogens. However, the mechanisms of how low salinity stress induces disease susceptibility remain poorly understood.

**Methods:**

We investigated the acute response of shrimp gut microbiota exposed to pathogens under low salinity stress. For comparison, shrimp were exposed to *Vibrio* infection under two salinity conditions: optimal salinity (Control group) and low salinity stress (Stress group). High throughput 16S rRNA sequencing and real-time PCR were employed to characterize the shrimp gut microbiota and quantify the severity level of *Vibrio* infection.

**Results:**

The results showed that low salinity stress increased *Vibrio* infection levels, reduced gut microbiota species richness, and perturbed microbial functions in the shrimp gut, leading to significant changes in lipopolysaccharide biosynthesis that promoted the growth of pathogens. Gut microbiota of the bacterial genera *Candidatus* Bacilliplasma, *Cellvibrio*, and *Photobacterium* were identified as biomarkers of the Stress group. The functions of the gut microbiota in the Stress group were primarily associated with cellular processes and the metabolism of lipid-related compounds.

**Conclusions:**

Our findings reveal how environmental stress, particularly low salinity, increases shrimp susceptibility to *Vibrio* infection by affecting the gut microbiota. This highlights the importance of avoiding low salinity stress and promoting gut microbiota resilience to maintain the health of shrimp.

**Supplementary Information:**

The online version contains supplementary material available at 10.1186/s12866-024-03407-0.

## Introduction

Aquaculture, the cultivation of valuable aquatic organisms under controlled or semi-natural conditions, is a rapidly expanding sector of the food industry. Contrasted with capture fishery, shrimp farming as a sustainable food production method plays a critical role in meeting the global growing demand for seafood and continues to expand its production volume. Since the 1950s, global aquaculture production has experienced rapid growth, whereas capture fishery production has not shown an increasing trend since the early 1990s [1]. In 2018, aquaculture production accounted for 52% of the total harvested weight of aquatic animals for human consumption [2]. It is projected that by 2050, aquaculture production will need to increase by nearly 60% over the 2018 level to meet the demand [1]. However, because most aquaculture systems are open with influent and effluent connected to natural waterways, climate change has become a significant threat to the quality and quantity of aquaculture production.

The effects of climate change, such as rising temperatures and increased frequency of extreme weather events, have a significant impact on aquatic environments. These changes in aquatic environments typically have a negative impact on aquaculture. For example, in Australia, during summer, the mortality of farmed abalone (*Haliotis laevigata*) reached 25% due to elevated water temperatures, resulting in a loss of AUD 1.75 million [3]. Similarly, as the water temperature rises, shrimp tend to stop feeding, often resulting in the mortality of both adult and larval shrimp [4]. Climate change has profound effects on rainfall intensity and variability. Heavy rainfall results in a substantial amount of freshwater entering aquaculture ponds, affecting the growth and development of farmed aquatic animals [5]. In addition, sudden shifts in salinity as a critical factor in disease outbreaks would likely affect the dynamics of microorganisms in the surrounding water and the susceptibility of aquatic animals to pathogen infection [6]. Abnormal rainfall causes sudden shifts in salinity, often triggering disease outbreaks in shrimp. For example, the outbreak of the White Spot Syndrome Virus (WSSV) coincided with the onset of the monsoon in Malaysia, during which intense rainfall reduced salinity levels in aquaculture ponds [7]. Highly variable and unpredictable environmental conditions caused by climate change increase the need for regular surveillance to maintain optimal water systems for each aquaculture species. More importantly, these environmental stresses threaten aquatic animal health directly by affecting host metabolic regulation or indirectly by increasing the risk of disease outbreaks [8].

*Litopenaeus vannamei*, commonly known as the Pacific white shrimp, is a dominant and valuable species in global aquaculture, with virtually all the production coming from aquaculture farming [9]. The Pacific white shrimp has a wide range of salinity tolerance from 1 to 50 ppt, with an optimum salinity around 20 ppt [10]. Semi-natural shrimp ponds are typically maintained at a salinity of 15–25 ppt [11], while the salinity can suddenly drop to 5–15 ppt after heavy rainfall [12, 13]. In the face of salinity stress, aquatic organisms are forced to make osmoregulatory adjustments by altering various enzymes and transporter proteins. These physiological adaptations require additional energy expenditure, which may increase their susceptibility to other stressors and diseases [14]. Previous studies have shown that variations in ambient salinity affect the growth performance and physiological responses of the Pacific white shrimp. The shrimp reared at 10 ppt (low salinity stress) showed a significant reduction in weight gain accompanied by a downregulation of metabolism-related genes when compared to shrimp reared at 20 ppt salinity [15]. When the salinity was drastically reduced to 10 ppt or less, the shrimp became more susceptible to viral infection, resulting in a mortality rate of up to 53.3% [16].

Aquaculture farmers have reported outbreaks of several diseases following heavy rains; for example, Acute Hepatopancreatic Necrosis Disease (AHPND) outbreaks are common in shrimp farming in Asia and Latin America [17, 18]. The mortality rate of shrimp infected with AHPND is rapid and high, resulting in annual economic losses exceeding one billion US dollars [19]. The primary pathogens of AHPND are unique strains of *Vibrio parahaemolyticus* (Vp). Non-AHPND-causing Vp is commonly present in aquatic environments as an opportunistic pathogen, whereas the AHPND-causing Vp strains possess unique virulence genes [20]. The Vp can be found in water and sediment (10^2^-10^4^ CFU per mL of water or g of sediment) and is also part of the commensal microbial community of many aquatic animals, including Pacific white shrimp [21–24]. In laboratory conditions, the Vp is able to thrive over a wide range of sodium chloride concentrations (between 0.5% and 10%), with an optimal concentration between 10 ppt and 30 ppt [25]. This range closely approximates the conditions of Pacific white shrimp aquaculture, making it difficult to prevent exposure of shrimp to Vp. Disease outbreaks are actually the result of interactions between hosts, pathogens, and the environment. Much evidence suggests that increased susceptibility to diseases might arise from the inability of osmotically stressed shrimp to generate a normal immune response [14]. However, the connection between these factors and disease outbreaks remains speculative.

For the treatment of bacterial diseases such as AHPND, the most common approach is to use antibiotics. However, the misuse of antibiotics has led to the selection and spread of antibiotic-resistant bacteria, which reduce the effectiveness of disease management and increasingly contribute to the potential ecological risks [26, 27]. Therefore, a new treatment approach by modulating the microbiota associated with aquaculture animals has been proposed, as it offers sustainable pathogen control methods [28]. The gut microbiota plays a critical role in several host physiological processes, including digestion, metabolism, and immune response [29, 30]. The gut microbiota influences nutrient absorption and energy regulation, and even manipulates host dietary behavior [31]. As shrimp lack adaptive immunity, the gut microbiota serves as an essential first line of defense against pathogen invasion and colonization [32]. Healthy gut microbiota can withstand low concentrations of pathogens through mechanisms like nutritional competition [33, 34]. Thus, the onset of disease can be attributed to environmental stress that causes dysbiosis in the gut microbiota, making it more susceptible to the invasion of pathogens [35]. In shrimp aquaculture, ponds with a background concentration (< 10^4^ CFU per mL of water) of pathogenic Vp rarely cause AHPND, with low or absent mortality [33]. However, low salinity stress may cause dysbiosis of the gut microbiota, increasing the opportunity for pathogenic Vp infection.

The mechanisms underlying disease susceptibility in shrimp due to low salinity stress remain unclear. Here, we aim to investigate the impact of low salinity stress on gut microbiota dysbiosis and shrimp susceptibility to *Vibrio* infection. Two experimental groups were set up: Control group: *Vibrio* infection under the optimal salinity (20 ppt), and Stress group: *Vibrio* infection under the stressful salinity level (10 ppt). We collected shrimp stomach samples at 6, 12, and 24 hpi to determine the levels of *Vibrio* infection and the characteristics of the gut microbiota, focusing on the early stress responses. Since the response of shrimp gut microbiota to environmental changes is rapid [36], determining the short-term changes after low salinity stress is required to understand the processes of gut microbiota dynamics in response to low salinity stress and pathogenic Vp infection. To reveal the specific differences in shrimp gut microbiota among experimental groups, high-throughput sequencing of the 16S rRNA gene was employed to characterize the shrimp gut microbiota. Compared to conventional culture-based methods, the 16S rRNA gene sequencing allows comprehensive profiling of microbial communities, revealing their diversity, composition, and functional potential [37]. We hypothesized that: low salinity stress decreases the diversity of shrimp gut microbiota and leads to a shift in composition towards the dominance of opportunistic pathogens, corresponding to the high infection level of pathogenic Vp.

## Materials and methods

### Pathogenic *Vibrio parahaemolyticus* strain

The AHPND-causing *Vibrio parahaemolyticus* strain (5HP) provided by Prof. Han-Ching Wang (National Cheng Kung University, Tainan, Taiwan) was isolated from the AHPND shrimp samples from Thailand [38]. The bacterial culture stock was stored in 25% glycerol at -80 °C prior to use.

### Experimental shrimp and water condition

Specific-pathogen-free (SPF) Pacific white shrimp (*Litopenaeus vannamei*) weighing between 2.0 ± 0.5 g from the Department of Aquaculture, National Pingtung University of Science and Technology (NPUST) were used for the experiments. All shrimp individuals were maintained in sterilized artificial seawater at a salinity of ~ 20 ppt and a temperature of 27 ± 1 ℃ for two days prior to the immersion challenge.

### Immersion and low-salinity stress challenges

Two experimental groups were conducted: Control group: immersion challenge with AHPND-causing *V. parahaemolyticus* 5HP strain under 20 ppt salinity, and Stress group: immersion challenge with AHPND-causing 5HP *Vibrio* strain under 10 ppt salinity. Challenge tests performed in duplicate for each group. 30 shrimp individuals were placed in a 30 L water tank, with 4 water tanks for experiments.

Bacterial cultures were recovered from frozen stocks on thiosulfate citrate bile salts sucrose agar (TCBS) plates. To prepare the bacteria inoculum, the colonies were subsequently inoculated into tryptic soy broth (TSB) medium containing 2% NaCl and incubated overnight at 30 °C with orbital shaking at 180 rpm [39]. The bacteria inoculum was scaled up to 100 ml and incubated at 30 °C for 1.5 h. The cell density of each bacterial culture (OD_600_) was then adjusted to 0.1–0.15 for immersion challenges. The shrimp were transferred into the mixture of 30 ml of bacterial suspension (10^7^ CFU/ml) and 30 L seawater in the tank, resulting in a final bacterial density of ~ 10^4^ CFU/ml per tank. The shrimp were kept under exposure condition for 24 h at 27 ± 0.5 ℃. At 6, 12 and 24 h post immersion (hereafter referred to as T06, T12 and T24), the entire stomachs of randomly selected shrimp were aseptically dissected and stored at -80 °C for DNA extraction. Four shrimp individuals were sampled from each tank at each time point, resulting in a cumulative total of 48 samples (4 tanks * 3 time points * 4 individuals) for subsequent analysis. The shrimp sampled for experiments were all alive at the time of sampling.

### Real-time PCR for AHPND detection

Genomic DNA from shrimp stomachs was extracted using QIAamp PowerFecal Pro DNA Kit (QIAGEN, Germany). The AHPND-related markers (AHPND plasmid and Toxin 1 gene) were screened by IQ REAL™ AHPND/EMS Quantitative System and the copies of shrimp genome were detected by IQ REAL™ WSSV Quantitative System (Gene Reach Biotechnology Corps, Taiwan) using TaqMan real-time PCR on CFX96 real-time system (Bio-Rad, USA). The kits contained artificial DNA comprising specific fragments of the AHPND plasmid and the PirAB^Vp^ gene (Toxin 1 gene), which were used as standards for constructing standard curves. In accordance with the methodology outlined by Chen et al. [40], a PCR amplification protocol utilizing two temperature stages was implemented. This involved a total of 40 cycles, with denaturation occurring at 93 °C for 15 s, followed by annealing and extension at 60 °C for 1 min. The copies of AHPND-related gene were normalized against the copies of shrimp genome in the stomach. Differences in the copy numbers of AHPND-associated genes over time were evaluated by Student’s t test or One-way analysis of variance (ANOVA) with Tukey test in the GraphPad Prism 8 software for Windows (GraphPad Software, USA, www.graphpad.com).

### 16S rRNA gene sequencing for gut microbiota profiling

To profile the shrimp gut microbiota, high-throughput sequencing of 16S rRNA gene amplicons was generated for analysis. Specifically, the hypervariable V4 region of the bacterial 16S rRNA gene was amplified by PCR, using a 515F-806R barcoded fusion primer set (515F: GTGYCAGCMGCCGCGGTAA; 806R: GGACTACNVGGGTWTCTAAT) [41]. PCR was performed with the gDNA from shrimp stomach and involved the following steps: initial denaturing at 95 °C for 3 min; 28 cycles of 95 °C for 30 s, 55 °C for 40 s, 72 °C for 50 s; final extension at 72 °C for 5 min. Amplicons in triplicate samples were pooled and purified using an AMPureXP PCR Purification Kit on a SPRIPlate 96 Super Magnet Plate (Agencourt, Brea, CA, USA) and quantified using a Qubit dsDNA HS Assay Kit on a Qubit 4.0 Fluorometer (Invitrogen, Carlsbad, CA, USA). The PCR product was purified and quantified using a Qubit dsDNA HS Assay Kit on a Qubit 4.0 Fluorometer (Invitrogen, Carlsbad, CA, USA). The pooled library with equal DNA concentration per sample was used for 2 × 300 bp paired-end sequencing. High-throughput sequencing was performed on the Illumina Miseq platform (BIOTOOLS, Taiwan).

### Processing of high-throughput sequencing data

Raw sequencing data of this study has been archived in the Sequence Read Archive (SRA) of the National Center for Biotechnology Information (NCBI) under the BioProject accession number PRJNA1018962. Sequencing data were processed using QIIME2 v. 2021.11 [42]. Raw reads were trimmed and non-biological sequences were removed by DADA2 standard filtering prior to further analysis [43]. The truncated sequences were merged to perform the amplicon sequence variant (ASV) method, and then non-chimeric ASV reads were generated. To generate a rooted phylogenetic tree, the representative sequences were extracted and processed using the phylogeny tool in QIIME2: align-to-tree-mafft-fast tree [44, 45]. Taxonomic classification of ASVs was performed at broad to fine levels, ranging from phylum to genus. This classification was achieved by employing the classify-sklearn method, which utilized a naïve Bayes classifier trained on Silva 138 99% operational taxonomic units (OTUs) derived from sequences within the 515F/806R region. (MD5: e05afad0fe87542704be96ff483824d4) [46–49]. All features annotated to bacterial taxa with phylum-level annotations were retained, but those containing either mitochondria or chloroplast in the taxonomic annotation were excluded. Subsequent analyses were based on a rarefied abundance table of features generated by DADA2, consisting of 9,939 sequences per sample.

### Analysis of the gut microbiota community

The rarefied community dataset included 48 samples, with *n* = 8 at each time point for both the Control and Stress groups. To assess the dissimilarities between the groups and examine the temporal changes of gut microbiota, alpha-diversity indices, including Chao1 and Shannon, were calculated by QIIME2 [42]. Visualization of alpha-diversity with box plots and Mann-Whitney tests were performed using GraphPad Prism 8 software for Windows (GraphPad Software, USA, www.graphpad.com). The significance level was set at *p* < 0.05. In addition, Venn diagrams were created based on the rarefied ASV table generated by QIIME2 to visualize the common (overlapping) and unique ASV numbers between the Control and Stress groups.

To assess the dissimilarities in gut microbiota composition (beta-diversity), the principal co-ordinates analysis (PCoA) based on the unweighted or weighted UniFrac distances was computed by QIIME2 [42], and visualized using the ggplot2 function of the ggplot R package [50] in R v4.2.0 [51]. Permutational multivariate analysis of variance (PERMANOVA) was performed using the vegan R package [52] to determine significant differences in the bacterial community composition between the Control and Stress groups. The significance level was set at *p* < 0.05. The ggplot2 R package was also employed to visualize the average relative abundances of several bacterial genera [50].

To predict functional abundances of the gut microbiota, the pipeline of Phylogenetic Investigation of Communities by Reconstruction of Unobserved States (PICRUSt2) was performed [53–56] in bioconda [57]. Specifically, the predicted functional units of PICRUSt2 were mapped to pathways and classified into three levels of functional categories based on the Kyoto Encyclopedia of Genes and Genomes (KEGG) database [58].

To identify the taxonomic or functional biomarkers that showed significant differences between the Control and Stress groups, the linear discriminative analysis Effect Size (LEfSe) [59] analysis was performed using lefse v1.0.8. post 1 [59] in the Python conda environment. The significance level for the Kruskal-Wallis test was set at *p* < 0.05, and the thresholds for linear discriminant analysis (LDA) scores were 3.0.

## Results

### Variation in *Vibrio* infection levels between control and stress groups

**Fig. 1:**
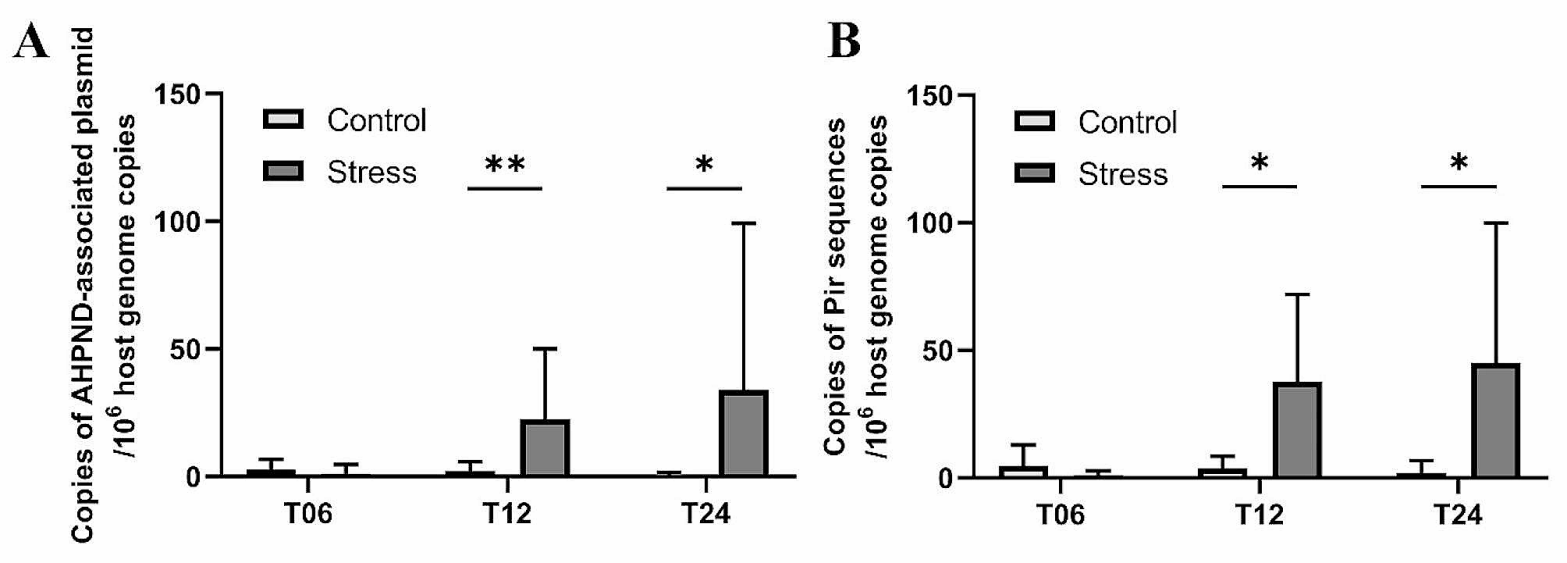
Variation in copy numbers of AHPND-associated plasmid and toxin gene between Control and Stress groups at each time point. The relative copies of (**A**) the AHPND plasmid and (**B**) the toxin gene were determined for individuals from the Control and Stress experimental groups at each time point (*n* = 8 per time point T06, T12, and T24). The relative copies of the AHPND plasmid and toxin gene were normalized to the host genome copies. Statistical significance was calculated using Student’s t-test (* *p* < 0.05, **p ≤ 0.01)

According to the real-time PCR results, the detected copies of the AHPND plasmid and toxin gene in the Stress group were approximately 10 times higher than those in the Control group (Fig. 1). The copy numbers of the AHPND plasmid (Fig. 1A) and toxin gene (Fig. 1B) were significantly higher in the Stress group than in the Control group at T12 and T24, whereas there was no significant difference at T06. Within each group, neither plasmid nor toxin gene showed differential expression over time (one-way ANOVA with Tukey’s post hoc test, Fig. S1).

### Differences in gut microbiota diversity between control and stress groups

**Fig. 2:**
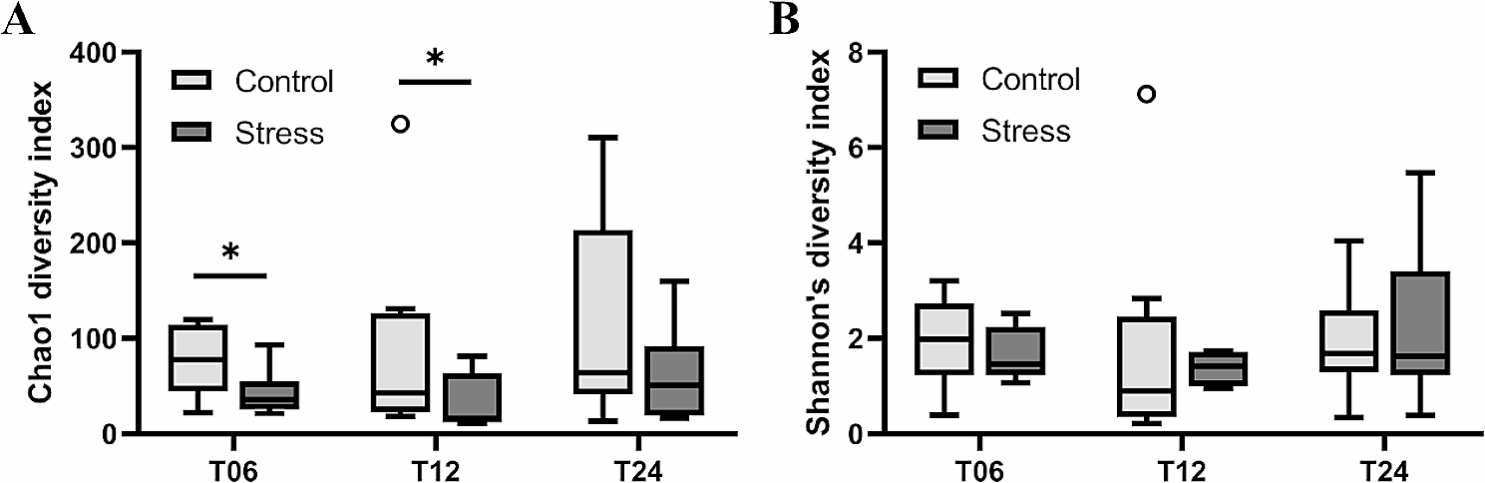
Differences in species diversity of gut microbiota between Control and Stress groups at each time point. Alpha-diversity indices of (**A**) Chao1 and (**B**) Shannon’s were determined in the Control and Stress groups at each time point (*n* = 8 per time point T06, T12, and T24). Statistical significance was calculated using the Mann-Whitney test (* *p* < 0.05)

**Fig. 3:**
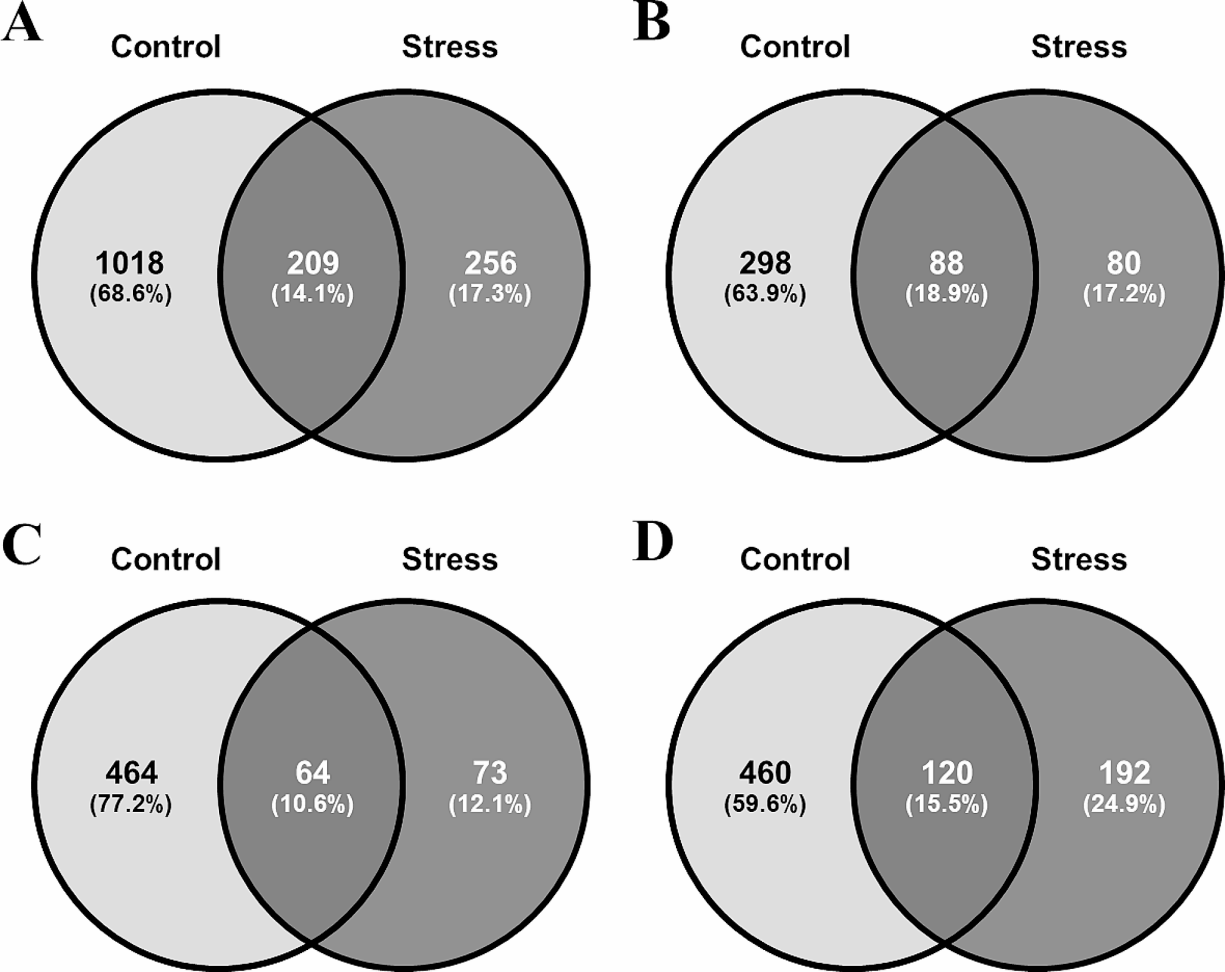
ASV compositions in Control and Stress groups. The Venn diagram shows the ASV compositions of the Control and Stress groups within (**A**) all time points or at (**B**) T06, (**C**) T12, (**D**) T24. The sample size was *n* = 8 at each time point

Regarding the α-diversity of the gut microbiota, the Stress group showed medially lower Chao1 values compared to the Control group at all time points, while only significant at T06 and T12 (Fig. 2A). However, no significant difference was found for Shannon index (Fig. 2B). Venn diagrams showed that the total number of unique ASVs was 1018 in the Control group and 256 in the Stress group, with only 209 ASVs in common between the two groups (Fig. 3A). Specifically, for all or at each time point (Fig. 3B and D), the proportion of unique ASVs was higher in the Control group (60–77%) than that in the Stress group (12–25%). In addition, the total number of ASVs in the Control group increased over time, whereas ASVs in the Stress group remained low and increased marginally atT24. These results suggest that the ASV richness of the shrimp gut microbiota could be reduced or limited by the low salinity stress.

### Differences in gut microbiota composition between control and stress groups

**Fig. 4:**
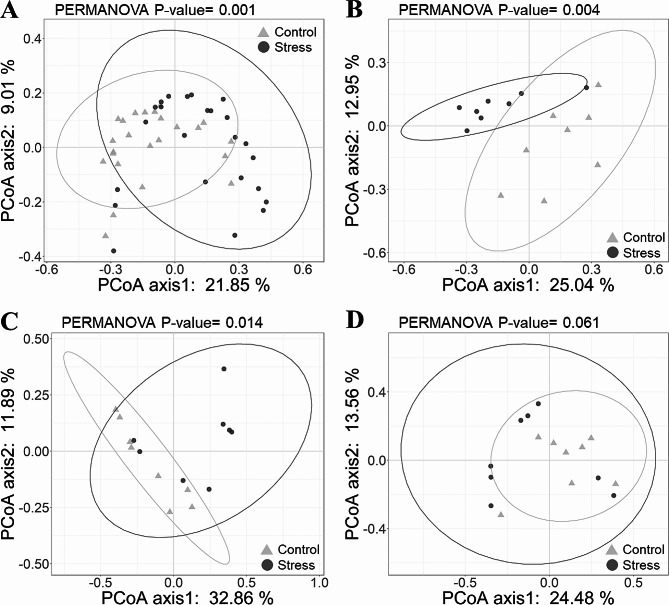
Principle coordinates analysis (PCoA) plots showing the dissimilarity of gut microbiota community composition based on the unweighted UniFrac distances. Each plot shows the pattern for (**A**) all time points, (**B**) T06, (**C**) T12, (**D**) T24 of the gut microbiota. Data points from Control and Stress experimental groups were labeled with different shapes and colors (Control = gray triangles, Stress = black dots). The sample size was *n* = 8 at each time point. Statistical significance was calculated using the PERMANOVA test

Principal coordinates analysis (PCoA) based on the unweighted UniFrac distances with PERMANOVA tests revealed differences in gut microbiota community composition between the Stress and Control groups (Fig. 4A), which were significant at T06 and T12 (Fig. 4B and C), but not at T24 (Fig. 4D). In contrast, for the PCoA based on the weighted UniFrac distances, a significant difference was found only at T24 (Fig. S2). These findings indicated that the separation of communities of different groups at T06 and T12 was driven by rare bacterial taxa, while at T24, it was driven by dominant taxa.

**Fig. 5:**
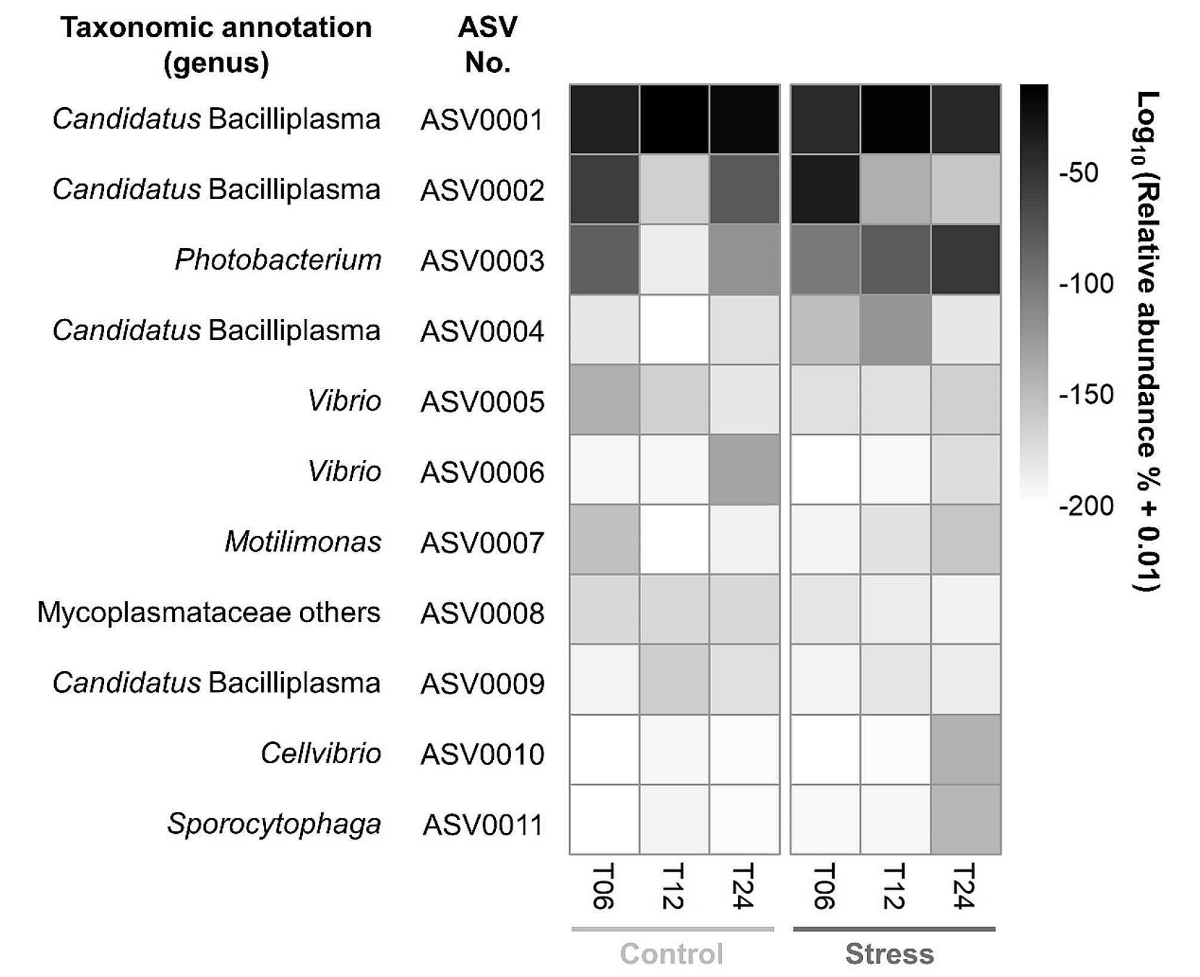
The relative abundances of the top dominant ASVs. Relative abundances of 11 ASVs (with mean abundance greater than 0.5%) were shown on average for each experimental group at three time points. The vertical axis showed the ASV number, corresponding to the numbers in Table S1. Color intensity signifies the relative abundance of respective ASV at each point in time

To identify the main contributors to the differences between the Control and Stress groups, we focused on the temporal dynamics of the top 11 dominant ASVs (Fig. 5). The top three ASVs cumulatively accounted for ~ 81% of the total abundance, as a good representation. The ASV0001 and ASV0002, belonging to *Candidatus* Bacilliplasma, were generally abundant in both groups. The ASV0001 was mainly enriched at T12, while the ASV0002 was mainly enriched at T06. The ASV0003, belonging to *Photobacterium*, increased over time in the Stress group. In addition, the ASV0005 abundance dynamics might represent the temporal colonization of the pathogenic Vp, since the representative sequence of the ASV0005 was 100% identical to the pathogenic Vp 5HP strain.

Focusing on the top three bacterial genera of the shrimp gut microbiota, *Candidatus* Bacilliplasma (40–91%) was the most dominant bacterial genus in both Control and Stress groups, followed by *Photobacterium* (1–29%) and *Vibrio* (1–4%) (Fig. S3). The relative abundance of *Candidatus* Bacilliplasma decreased with time in the Stress group (Fig. S3B), while it remained dominant in the Control group (Fig. S3A). The relative abundance of *Photobacterium* increased with time in the Stress group (Fig. S3B), while it decreased at T12 and T24 in the Control group (Fig. S3A). The relative abundance of *Vibrio* remained in a constant ratio over time in both groups (Fig. S3).

### Taxonomic biomarkers between control and stress groups

**Fig. 6:**
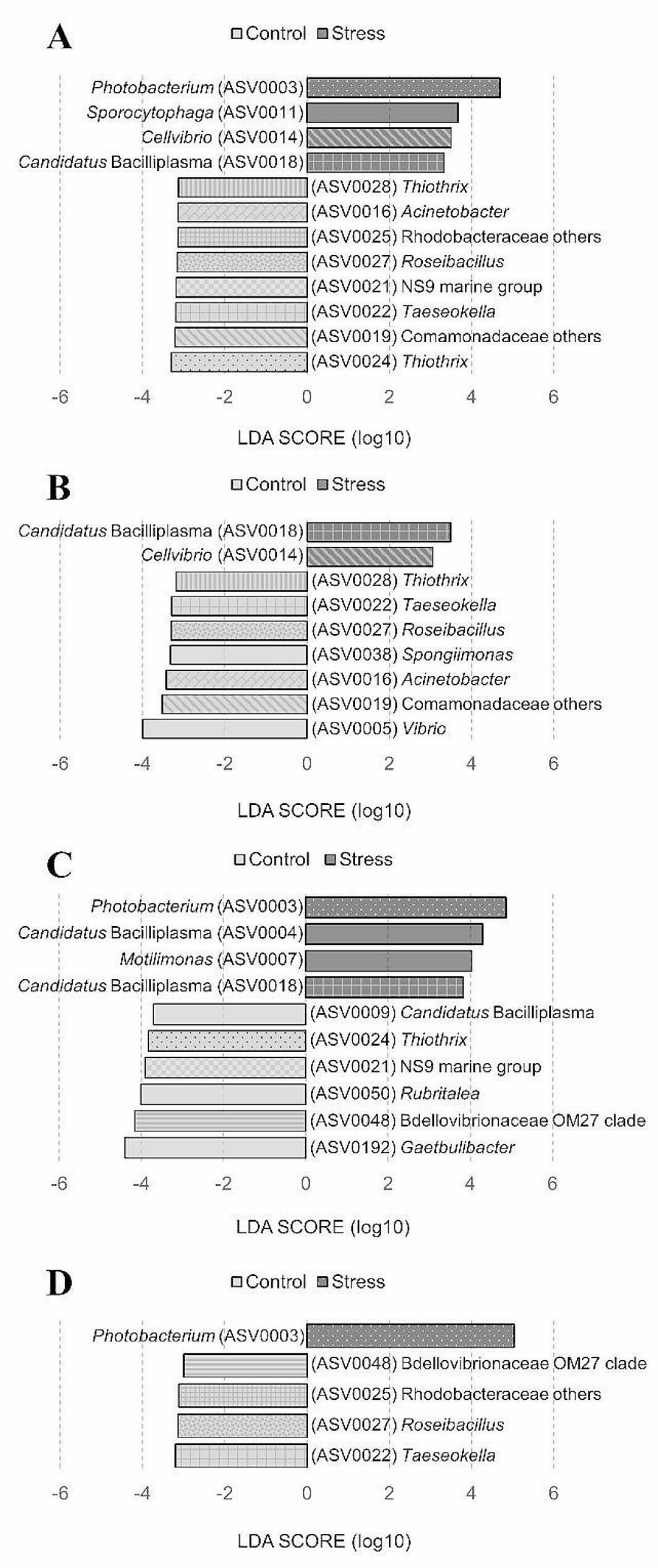
LEfSe showing taxonomic biomarkers (ASVs) that differed in abundance between Control and Stress groups. Identification of gut microbiota ASVs that differentiated the two groups for (**A**) all time points, (**B**) T06, (**C**) T12, and (**D**) T24 by LDA effect size. The ASV ID number (here with annotation at the genus level) corresponded to the numbers shown in Table S2. The pattern-coded bars represent ASVs that reoccur at different time points. The differences were significant (*p* < 0.05) among classes (Kruskal-Wallis test). The threshold value for the logarithmic LDA score was 3.0

To identify the taxonomic biomarkers characterizing inter-group differences, linear discriminant analysis effect size (LEfSe) analysis was performed at the ASV level (Fig. 6). For all or each time point, the number of detected biomarkers in the Control group was approximately twice that of the Stress group. For all time points, ASV0024 classified as *Thiothrix*, ASV0019 classified as Comamonadaceae others, and ASV0022 classified as *Taeseokella* were identified biomarkers with the highest LDA scores for the Control group (Fig. 6A). At T06, ASV0018 classified as *Candidatus* Bacilliplasma and ASV0014 classified as *Cellvibrio* were the identified biomarkers with the highest LDA scores for the Stress group (Fig. 6B). At T12 and T24, ASV0003 classified as *Photobacterium* consistently emerged as the highest-scoring biomarker for the Stress group (Fig. 6C and D).

### Functional biomarkers between control and stress groups

**Fig. 7:**
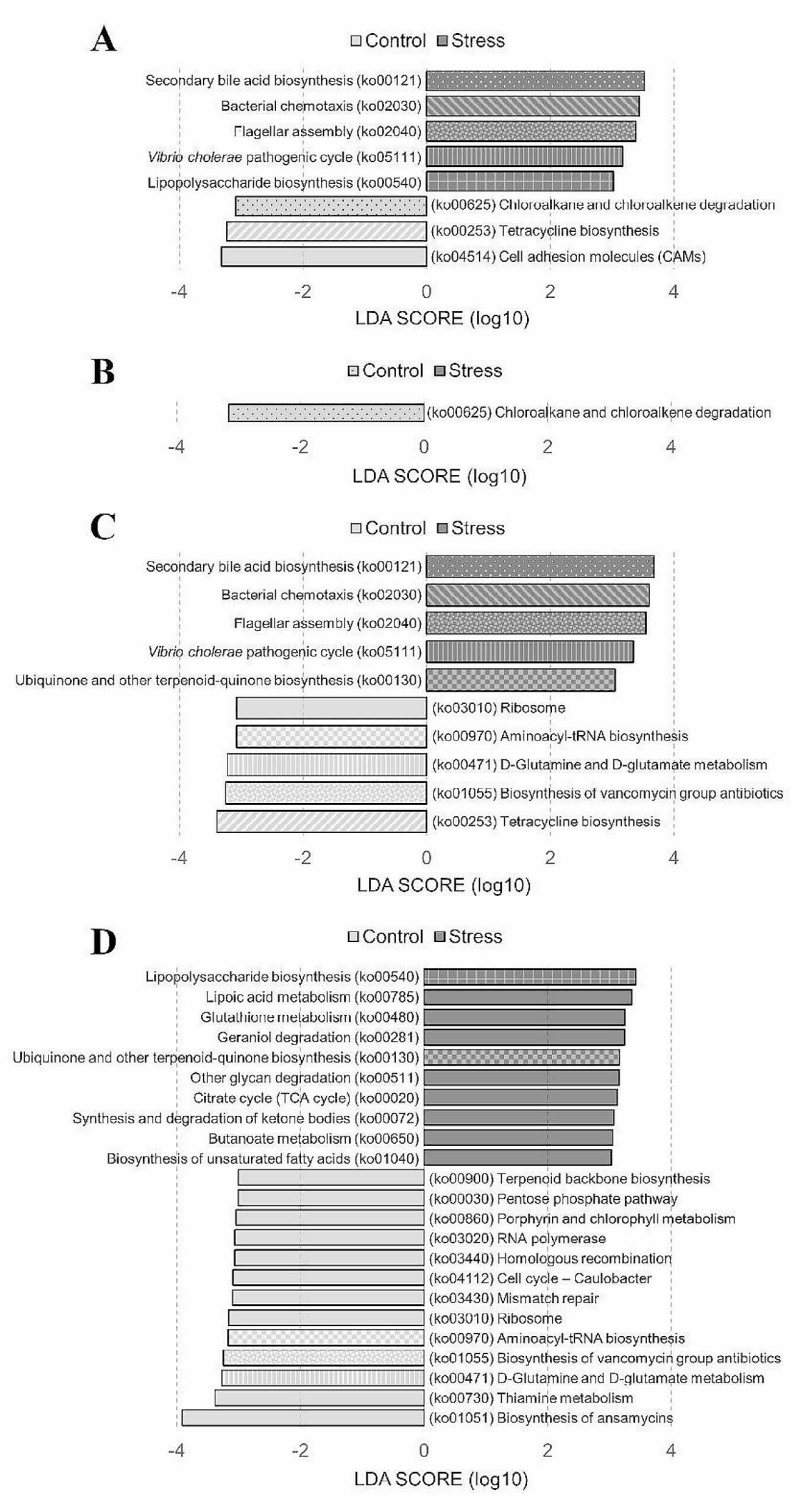
LEfSe showing functional biomarkers that differed in abundance between Control and Stress groups. Identification of predicted gut microbiota functions that differentiated the two groups for (**A**) all time points, (**B**) T06, (**C**) T12, and (**D**) T24 by LDA effect size. The pattern-coded bars represent functions that reoccur at different time points. The functional biomarkers (here with annotation at the KEGG 3 level) corresponded to the predicted microbial pathways in Table S3. The differences were significant (*p* < 0.05) among classes (Kruskal-Wallis test). The threshold value of the logarithmic LDA score was 3.0

To identify the predicted functional biomarkers characterizing inter-group differences, linear discriminant analysis effect size (LEfSe) analysis was performed with the PICRUSt2-predicted pathways based on the KEGG database (Fig. 7). For all time points, secondary bile acid biosynthesis (ko00121) and bacterial chemotaxis (ko02030) and flagellar assembly (ko02040) were enriched in the Stress group (Fig. 7A). At T06 (Fig. 7B), only one differentially abundant pathway was detected, namely, chloroalkane and chloroalkene degradation (ko00625), which was enriched in the Control group. At T12 (Fig. 7C), tetracycline biosynthesis (ko00253) and biosynthesis of vancomycin group antibiotics (ko01055) were enriched with the highest LDA score in the Control group. In comparison, secondary bile acid biosynthesis (ko00121) and bacterial chemotaxis (ko02030) were enriched with the highest LDA score in the Stress group. Moreover, *Vibrio cholerae* pathogenic cycle as a pathogen specific pathway emerged at T12 in the Stress group (Fig. 7C). At T24 (Fig. 7D), biosynthesis of ansamycins (ko01051) and thiamine metabolism (ko00730) were enriched with the highest LDA score in the Control group. While lipopolysaccharide biosynthesis (ko00540) and lipoic acid metabolism (ko00785) were enriched with the highest LDA score in the Stress group. Overall, the Control group exhibited diverse functions, mainly associated with environmental information processing, genetic information processing, and various metabolic processes. In the Stress group, signature functions were mainly associated with cellular processes and the metabolism of lipid-related compounds (Table S3).

## Discussion

### Low salinity stress increases the risk of *Vibrio parahaemolyticus* infection

The real-time PCR results showed a significant increase in the copy numbers of AHPND plasmid and toxin gene of pathogenic Vp in the Stress group (Fig. 1). However, the relative abundance of *Vibrio* in shrimp gut microbiota did not differ significantly between the Control and Stress groups (Fig. 5 and Fig. S3). The higher copy number of AHPND-associated genes suggests that the virulence of pathogenic Vp may be regulated under low salinity stress, as salinity has previously been shown to alter PirA gene expression and regulate Vp AHPND virulence [60]. Furthermore, the unchanged abundance of *Vibrio* suggests that the pathogenic Vp may replace the non-pathogenic Vp due to adaptive selection. It is also important to note that the pVA1 plasmid carried by the pathogenic Vp can be transferred to some non-pathogenic Vp [61]. These could potentially exacerbate the disease without increasing the abundance of *Vibrio*.

The copy numbers of AHPND plasmid and toxin gene in the Stress group increased significantly at T12 and T24, while at T06, although the trend showed higher copy numbers in the Stress group compared to the Control group, the difference was not significant (Fig. 1). In our previous research [62], the highest detection of AHPND plasmid and toxin gene occurred at 12 hpi, whereas in this study, the highest detection appeared at both 12 and 24 hpi (Fig. S1). These observations may reflect the extended adaptation process of pathogenic Vp to low salinity stress. Under stressful conditions, the pathogenic Vp is able to modulate its gene expression and metabolic pathways to adapt to the changing environment in the shrimp gut, thereby regulating its virulence [63, 64]. More importantly, the toxin secretion systems of pathogenic Vp can mediate inter- and intra-species competition, influencing the diversity and composition of shrimp gut microbiota [65].

### Low salinity stress reduces gut microbiota diversity in shrimp

The impact of low salinity stress on the α-diversity of shrimp gut microbiota has become a prominent research focus, as microbial diversity often reflects the health status of the gut microbiota [40]. In the Stress group, the Chao1 values were significantly reduced at T06 and T12 (Fig. 2A), indicating a decrease in species richness of the shrimp gut microbiota during the early stage of infection under low salinity stress. As time progressed, at T24, no significant difference was observed between the Stress and Control groups, which is consistent with the results observed in black tiger shrimp exposed to *Vibrio harveyi* [66]. However, considering the Shannon index, no significant differences between groups were observed at all time points (Fig. 2B), suggesting that although some species declined or disappeared due to the salinity stress, the relative distribution of dominant to rare species remained unaffected. This could be attributed to the differential adaptability of microbial species to low salinity stress, where core populations may have the ability to tolerate salinity fluctuations and thus maintain a relatively stable abundance and distribution [67].

In addition, the Venn diagrams showed the number of unique and shared ASVs between the Stress and Control groups (Fig. 3). The proportion of unique ASVs in the Control group was significantly higher than that in the Stress group, further indicating that the shrimp gut microbiota possessed higher species diversity under non-stressed conditions. The reduction in microbial diversity under stress could potentially affect gut functionality and shrimp health. The increasing trend in the total number of ASVs in the Control group over time also piqued our interest, suggesting that the gut microbiota in the Control group exhibited dynamic and diverse characteristics even in the presence of pathogenic bacteria, showing a certain degree of resilience and ability to maintain its diversity [34]. In contrast, the low number of ASVs in the Stress group at T24 indicated that the shrimp gut microbiota might exhibit delayed or unsuccessful adaptation to low salinity stress, leading to gut microbiota dysbiosis and potential bacterial community disruption.

### Low salinity stress alters gut microbiota composition in shrimp

Our results show that low salinity stress has a visible effect on the composition of the gut microbiota. At T06 and T12, the compositional changes are mainly driven by shifts in rare bacterial taxa (Fig. 4), whereas at T24, the changes are mainly influenced by dominant bacterial taxa (Fig. S2). It has been suggested that rare taxa may be more sensitive to changes in salinity and other environmental stresses than abundant taxa [68]; thus, the response of microbial communities to perturbations is often determined by rare bacterial taxa [69]. Abundant taxa can utilize a wide range of resources, making them more resistant to extinction and easier to disperse. In addition, rare taxa may occupy less suitable microecological niches, making them more vulnerable to environmental changes, including salinity stress [70]. Moreover, the alterations in gut microbiota composition may create opportunities for the invasion or spread of opportunistic pathogens, which in turn disrupt the cooperative interactions among resident species [29]. Specifically, in this study, the dominant bacterial taxa in the Stress group shifted toward opportunistic pathogens, including *Candidatus* Bacilliplasma, *Photobacterium*, and *Cellvibrio* (Fig. 6B and C, and 6D).

*Candidatus* Bacilliplasma has been reported to be prevalently dominant in the gastrointestinal tract of shrimp [71]. In this study, several ASVs belonging to *Candidatus* Bacilliplasma showed rich abundances (Fig. 5) and were identified as biomarkers for either the Stress or Control group (Fig. 6), indicating the high phylogenetic diversity and distinct ecological characteristics within this genus. Some *Candidatus* Bacilliplasma strains have been proposed as opportunistic pathogens in shrimp [71], while some strains have been suggested as probiotics [72]. In the shrimp gut, various strains of *Candidatus* Bacilliplasma could interact differently with pathogenic *Vibrio* strains, either enhancing or inhibiting infections [40].

*Photobacterium* was present in both Stress and Control groups, but was specially selected as a biomarker for the Stress group at T12 and T24 (Fig. 6). The increased abundance of the *Photobacterium* in the Stress group could be attributed to the specific niches created by the infection of pathogenic Vp at T12 and T24, consistent with the detection period of AHPND-related genes [73]. *Vibrio parahaemolyticus* infections may trigger the growth of other potential pathogenic bacteria, resulting in a shift in microbial composition towards the dominance of opportunistic pathogens [74]. *Photobacterium* is an opportunistic pathogen that belongs to Vibrionaceae as the same as *Vibrio*. Vibrionaceae has been served as a signature for the diagnosis of AHPND [75].

*Cellvibrio* was identified as a biomarker for the Stress group at T06 (Fig. 6). *Cellvibrio* is known for the abundance of carbohydrate-active enzymes (CAZymes) encoded in its genome [76]. Among these, lytic polysaccharide monooxygenases (LPMOs) involved in chitin degradation have been characterized [77]. LPMOs have been shown to play a role in chitin degradation and virulence in several pathogens [78, 79]. For example, LPMOs contribute to the pathogenicity during the invasion stage of cold-water vibriosis (CWV) [80]. Thus, chitin-degrading enzymes may serve not only nutritional acquisition but also protection against host defense mechanisms for bacteria. In addition, one study has shown that *Cellvibrio* abundance is positively correlated with changes in polysaccharide metabolism while negatively correlated with changes in immune-related genes [81]. In this study, *Cellvibrio* may facilitate the invasion of pathogenic Vp at an early stage. Currently, information on *Cellvibrio* in the shrimp gut microbiota is relatively limited, and further research is warranted.

*Vibrio*, on the other hand, remained relatively low in abundance in both Stress and Control groups. Low salinity stress led to an increase in the expression of virulence genes (Fig. 1), but not to an increase in the colonization of pathogenic Vp (Fig. S3). This may indicate that the disease susceptibility induced by low salinity stress is not necessarily related to the abundance of pathogenic bacteria, but rather to the regulation of virulence factors [20]. Moreover, the PirAB^Vp^ toxin secreted by pathogenic Vp could modulate the virulence of non-pathogenic *Vibrio* and exacerbate vibriosis [82], which has systemic effects on gut functionality.

In our previous study, we compared the gut microbiota of healthy shrimp with that of shrimp infected with AHPND without salinity stress [62]. The biomarkers in diseased shrimp belonged to *Photobacterium* and *Vibrio*, whereas the biomarkers in healthy shrimp belonged to *Candidatus* Bacilliplasma. The abundance of *Photobacterium* often increased significantly with infection of highly virulent *Vibrio* [83]. In the results of this study, the abundance of *Photobacterium* was significantly higher in the Stress group compared to the Control group (Fig. 6), suggesting that low salinity stress further exacerbates AHPND.

### Low salinity stress modulates gut microbiota functions in shrimp

Our analysis indicated that the gut microbiota of shrimp infected with AHPND showed different functions under different salinity conditions. Low salinity stress would alter the functions of shrimp gut microbiota. At T06 (Fig. 7B), a significant difference in the degradation of chloroalkane and chloroalkene was detected between the two groups. Chloroalkanes and chloroalkenes are xenobiotics found in aquatic environments [84], suggesting a reduced ability of shrimp gut bacteria to eliminate xenobiotic compounds under low salinity stress [85]. As the infection progressed at T12 and T24 (Fig. 7C and D), pathways associated with antibiotic synthesis (such as tetracycline, ansamycin, and vancomycin) were significantly downregulated in the Stress group, while pathways associated with bacterial survival (such as flagellar assembly) were significantly upregulated. This shift in the functionality of the Stress group indicates a tendency to favor the growth of pathogens [86]. Specifically, at T12, secondary bile acid biosynthesis, bacterial chemotaxis, flagellar assembly, and *Vibrio cholerae* pathogenic cycle were significantly upregulated. Bile acids positively influenced the formation of pathogenic Vp biofilms and toxin secretion [87]. Previous studies have suggested that biofilm formation can assist pathogens in resource acquisition and protection from chemical or predatory pressures [88]. In addition, virulence factors can directly attack host cells, induce host inflammatory responses, and thereby create suitable ecological niches for pathogen invasion [89]. Furthermore, in the Stress group, D-glutamine and D-glutamate metabolism were inhibited, possibly due to increased energy demands caused by osmotic stress from low salinity stress, leading to reduced physiological biosynthesis [90]. Moreover, at T24, in the Stress group, the lipopolysaccharide biosynthesis and lipoic acid metabolism were significantly upregulated (Fig. 7D). Lipopolysaccharides are major components of the outer membranes of Gram-negative bacteria [91], which may reflect the colonization and expansion of pathogenic Vp. In addition, lipopolysaccharide (LPS) in the cell walls of Gram-negative bacteria has been predicted to disrupt junctional complexes and increase intestinal permeability and inflammation [92]. Lipoic acid metabolism is a well-known metabolic pathway in the shrimp gut, the significant upregulation of which may be attributed to the replication metabolic and biosynthetic demands of pathogens [93]. These suggest that the exacerbation of AHPND and severe gut lesions in the Stress group. Low salinity stress enhanced the infection of AHPND, leading to further changes in gut microbiota functions.

### Interaction between low salinity stress, gut microbiota and shrimp immune during AHPND

In this study, we aimed to investigate the relationship between salinity stress, changes in the shrimp gut microbiota, and susceptibility to AHPND. While our results suggest an association between changes in the gut microbiome and increased susceptibility to AHPND, impairment of the host immune system may also be a potential factor. Several studies have shown that low salinity stress has a significant impact on the immune system of shrimp. Under low salinity stress, shrimp immune parameters decrease, resulting in reduced resistance to pathogens [94–95]. In addition, low salinity environments may also affect the shrimp gut microbiota, with lower diversity and simpler structure [6]. These suggest that low salinity stress may influence shrimp disease susceptibility through two pathways: on the one hand, low salinity stress may weaken the immune system of shrimp, making them more susceptible to pathogen infection; on the other hand, changes in the gut microbiota caused by low salinity stress may also promote the colonization of pathogenic bacteria.

Moreover, there is a complex relationship between host immunity and gut microbiota. Increasing evidence supports the view that the microbiota plays an important role in regulating the host immune system. Microbiota influences host immune responses during both health and disease. On the one hand, studies indicate that germ-free animals show broad developmental defects in the immune system [96], which can be rescued after the introduction of gut bacteria [97], suggesting an interrelationship between the immune system and the microbiota. On the other hand, several potential pathogens are part of the normal gut microbiota that generally do not cause disease [98]. This may be due to the antagonism of commensal bacteria in healthy shrimp that inhibits pathogen overgrowth and virulence expression [99]. For pathogenic Vp, successful infection requires initial entry and continued colonization in the stomach, where the gut microbiota serves as the first line of defense in the host immune system, highlighting the importance of the gut microbiota.

### Enhancing gut microbiota resilience is needed for healthy shrimp production

Our findings reveal how environmental stress, particularly low salinity, increases shrimp susceptibility to pathogenic Vp infection by affecting the gut microbiota. Maintaining a high level of environmental quality is of paramount importance in aquaculture. In fact, many aquaculture operations have a negative impact on the local environment through the misuse of chemicals, the discharge of wastes, and the transmission of diseases, posing a threat to the sustainability of aquaculture [100]. In addition, multiple ecological feedback loops link human health and seafood production, with aquaculture playing a pivotal role in food security in many regions of the world [101, 102]. Therefore, the implementation of the “One Health” concept in aquaculture is essential to ensure the integrated health of the environment, farmed animals, and humans [27].

For aquaculture animals, because opportunistic pathogens are constantly stored in the aquatic environment, the risk of pathogen reinfection remains high even after complete environmental disinfection. Disease management strategies therefore require a paradigm shift towards promoting system resilience rather than pathogen eradication [103]. This highlights the importance of avoiding low salinity stress and promoting gut microbiota resilience to maintain the health of aquaculture shrimp. Due to environmental stress, gut microbiota would fluctuate and transition from a healthy to an unstable state [34]. Resilient gut microbiota communities are able to return to a healthy state, whereas non-resilient communities would shift to an unhealthy state. Low salinity stress could cause the shrimp gut microbiota to become unstable, creating an opportunity for invasion by pathogenic Vp. In addition to avoiding the occurrence of environmental stress, promoting the resilience of the gut microbiota could be a way to prevent critical shifts toward dysbiosis. The biomarkers detected in the Control group may help to characterize resilient gut microbiota communities and hold promising potential as probiotics (Fig. 6). Probiotic intervention has been proposed as an environmentally friendly and sustainable approach to restore a healthy gut microbiota in viral and bacterial shrimp diseases [104]. Probiotics could suppress opportunistic pathogens by stimulating biodiversity [105] and competitive exclusion [106], thereby aiding in restoring symbiotic microbiomes.

In conclusion, this study investigated the acute response of shrimp gut microbiota exposed to pathogens under environmental stress and revealed the potential mechanism of low salinity stress in enhancing disease susceptibility (Fig. S4). The decrease in species richness and changes in composition reflect possible responses in the community structure of the gut microbiota under environmental stress. The stability of the gut microbiota was disrupted by low salinity stress, consequently enhancing shrimp susceptibility to the infection of pathogenic *Vibrio parahaemolyticus* (Vp). These findings contribute to understanding the intricate interplay between environmental stress, gut microbiota, and potential disease outbreaks, providing valuable insights for shrimp health management.

### Electronic supplementary material

Below is the link to the electronic supplementary material.

Supplementary Material 1

Supplementary Material 2

## Data Availability

Raw sequencing data of this study has been archived in the Sequence Read Archive (SRA) of the National Center for Biotechnology Information (NCBI) under the BioProject accession number PRJNA1018962 (https://www.ncbi.nlm.nih.gov/sra/?term=PRJNA1018962).
