# Supplementary material for: Low salinity stress increases the risk of Vibrio parahaemolyticus infection and gut microbiota dysbiosis in Pacific white shrimp
Source: BMC Microbiol. 2024 Jul 25;24:275. doi: 10.1186/s12866-024-03407-0 (PMC11271031; doi:10.1186/s12866-024-03407-0)
Supplement: Supplementary file 1 — Supplementary Material 1 [file 12866_2024_3407_MOESM1_ESM.docx]

**Table S1. Annotation of the top 10 ASVs in shrimp gut microbiota.**

| **ASV No.** | **Average abundance (%)** | **Taxonomic annotation (phylum to genus)** |
| --- | --- | --- |
| ASV0001 | 53.70 | d__Bacteria; p__Firmicutes; c__Bacilli; o__Mycoplasmatales; f__Mycoplasmataceae; g__Candidatus_Bacilloplasma |
| ASV0002 | 15.54 | d__Bacteria; p__Firmicutes; c__Bacilli; o__Mycoplasmatales; f__Mycoplasmataceae; g__Candidatus_Bacilloplasma; s__uncultured_bacterium |
| ASV0003 | 12.06 | d__Bacteria; p__Proteobacteria; c__Gammaproteobacteria; o__Vibrionales; f__Vibrionaceae; g__Photobacterium; s__Photobacterium_damselae |
| ASV0004 | 1.47 | d__Bacteria; p__Firmicutes; c__Bacilli; o__Mycoplasmatales; f__Mycoplasmataceae; g__Candidatus_Bacilloplasma; s__uncultured_bacterium |
| ASV0005 | 1.24 | d__Bacteria; p__Proteobacteria; c__Gammaproteobacteria; o__Vibrionales; f__Vibrionaceae; g__Vibrio |
| ASV0006 | 0.82 | d__Bacteria; p__Proteobacteria; c__Gammaproteobacteria; o__Vibrionales; f__Vibrionaceae; g__Vibrio |
| ASV0007 | 0.77 | d__Bacteria; p__Proteobacteria; c__Gammaproteobacteria; o__Alteromonadales; f__Psychromonadaceae; g__Motilimonas; s__uncultured_bacterium |
| ASV0008 | 0.69 | d__Bacteria; p__Firmicutes; c__Bacilli; o__Mycoplasmatales; f__Mycoplasmataceae; g__uncultured; s__uncultured_Firmicutes |
| ASV0009 | 0.57 | d__Bacteria; p__Firmicutes; c__Bacilli; o__Mycoplasmatales; f__Mycoplasmataceae; g__Candidatus_Bacilloplasma |
| ASV0010 | 0.52 | d__Bacteria; p__Proteobacteria; c__Gammaproteobacteria; o__Cellvibrionales; f__Cellvibrionaceae; g__Cellvibrio; s__uncultured_bacterium |
| ASV0011 | 0.52 | d__Bacteria; p__Bacteroidota; c__Bacteroidia; o__Cytophagales; f__Cytophagaceae; g__Sporocytophaga; s__uncultured_bacterium |

**Table S2. LEfSe discovery of taxonomic biomarkers (ASVs with phylum to genus annotation).**

| **Time point** | **ASV No.** | **Taxonomic annotation (phylum to genus)** | **Biomarkers (group)** |
| --- | --- | --- | --- |
| All | ASV0003 | d__Bacteria; p__Proteobacteria; c__Gammaproteobacteria; o__Vibrionales; f__Vibrionaceae; g__Photobacterium; s__Photobacterium_damselae | Stress |
|  | ASV0011 | d__Bacteria; p__Bacteroidota; c__Bacteroidia; o__Cytophagales; f__Cytophagaceae; g__Sporocytophaga; s__uncultured_bacterium | Stress |
|  | ASV0014 | d__Bacteria; p__Proteobacteria; c__Gammaproteobacteria; o__Cellvibrionales; f__Cellvibrionaceae; g__Cellvibrio | Stress |
|  | ASV0018 | d__Bacteria; p__Firmicutes; c__Bacilli; o__Mycoplasmatales; f__Mycoplasmataceae; g__Candidatus_Bacilloplasma; s__uncultured_bacterium | Stress |
|  | ASV0028 | d__Bacteria; p__Proteobacteria; c__Gammaproteobacteria; o__Thiotrichales; f__Thiotrichaceae; g__Thiothrix | Control |
|  | ASV0016 | d__Bacteria; p__Proteobacteria; c__Gammaproteobacteria; o__Pseudomonadales; f__Moraxellaceae; g__Acinetobacter | Control |
|  | ASV0025 | d__Bacteria; p__Proteobacteria; c__Alphaproteobacteria; o__Rhodobacterales; f__Rhodobacteraceae | Control |
|  | ASV0027 | d__Bacteria; p__Verrucomicrobiota; c__Verrucomicrobiae; o__Verrucomicrobiales; f__Rubritaleaceae; g__Roseibacillus; s__uncultured_Verrucomicrobia | Control |
|  | ASV0021 | d__Bacteria; p__Bacteroidota; c__Bacteroidia; o__Flavobacteriales; f__NS9_marine_group; g__NS9_marine_group; s__uncultured_Flavobacterium | Control |
|  | ASV0022 | d__Bacteria; p__Bacteroidota; c__Bacteroidia; o__Cytophagales; f__Spirosomaceae; g__Taeseokella | Control |
|  | ASV0019 | d__Bacteria; p__Proteobacteria; c__Gammaproteobacteria; o__Burkholderiales; f__Comamonadaceae | Control |
|  | ASV0024 | d__Bacteria; p__Proteobacteria; c__Gammaproteobacteria; o__Thiotrichales; f__Thiotrichaceae; g__Thiothrix | Control |
| T06 | ASV0018 | d__Bacteria; p__Firmicutes; c__Bacilli; o__Mycoplasmatales; f__Mycoplasmataceae; g__Candidatus_Bacilloplasma; s__uncultured_bacterium | Stress |
|  | ASV0014 | d__Bacteria; p__Proteobacteria; c__Gammaproteobacteria; o__Cellvibrionales; f__Cellvibrionaceae; g__Cellvibrio | Stress |
|  | ASV0028 | d__Bacteria; p__Proteobacteria; c__Gammaproteobacteria; o__Thiotrichales; f__Thiotrichaceae; g__Thiothrix | Control |
|  | ASV0022 | d__Bacteria; p__Bacteroidota; c__Bacteroidia; o__Cytophagales; f__Spirosomaceae; g__Taeseokella | Control |
|  | ASV0027 | d__Bacteria; p__Verrucomicrobiota; c__Verrucomicrobiae; o__Verrucomicrobiales; f__Rubritaleaceae; g__Roseibacillus; s__uncultured_Verrucomicrobia | Control |
|  | ASV0038 | d__Bacteria; p__Bacteroidota; c__Bacteroidia; o__Flavobacteriales; f__Flavobacteriaceae; g__Spongiimonas; s__uncultured_Ornithobacterium | Control |
|  | ASV0016 | d__Bacteria; p__Proteobacteria; c__Gammaproteobacteria; o__Pseudomonadales; f__Moraxellaceae; g__Acinetobacter | Control |
|  | ASV0019 | d__Bacteria; p__Proteobacteria; c__Gammaproteobacteria; o__Burkholderiales; f__Comamonadaceae | Control |
|  | ASV0005 | d__Bacteria; p__Proteobacteria; c__Gammaproteobacteria; o__Vibrionales; f__Vibrionaceae; g__Vibrio | Control |
| T12 | ASV0003 | d__Bacteria; p__Proteobacteria; c__Gammaproteobacteria; o__Vibrionales; f__Vibrionaceae; g__Photobacterium; s__Photobacterium_damselae | Stress |
|  | ASV0004 | d__Bacteria; p__Firmicutes; c__Bacilli; o__Mycoplasmatales; f__Mycoplasmataceae; g__Candidatus_Bacilloplasma; s__uncultured_bacterium | Stress |
|  | ASV0007 | d__Bacteria; p__Proteobacteria; c__Gammaproteobacteria; o__Alteromonadales; f__Psychromonadaceae; g__Motilimonas; s__uncultured_bacterium | Stress |
|  | ASV0018 | d__Bacteria; p__Firmicutes; c__Bacilli; o__Mycoplasmatales; f__Mycoplasmataceae; g__Candidatus_Bacilloplasma; s__uncultured_bacterium | Stress |
|  | ASV0009 | d__Bacteria; p__Firmicutes; c__Bacilli; o__Mycoplasmatales; f__Mycoplasmataceae; g__Candidatus_Bacilloplasma | Control |
|  | ASV0024 | d__Bacteria; p__Proteobacteria; c__Gammaproteobacteria; o__Thiotrichales; f__Thiotrichaceae; g__Thiothrix | Control |
|  | ASV0021 | d__Bacteria; p__Bacteroidota; c__Bacteroidia; o__Flavobacteriales; f__NS9_marine_group; g__NS9_marine_group; s__uncultured_Flavobacterium | Control |
|  | ASV0050 | d__Bacteria; p__Verrucomicrobiota; c__Verrucomicrobiae; o__Verrucomicrobiales; f__Rubritaleaceae; g__Rubritalea | Control |
|  | ASV0048 | d__Bacteria; p__Bdellovibrionota; c__Bdellovibrionia; o__Bdellovibrionales; f__Bdellovibrionaceae; g__OM27_clade; s__uncultured_delta | Control |
|  | ASV0192 | d__Bacteria; p__Bacteroidota; c__Bacteroidia; o__Flavobacteriales; f__Flavobacteriaceae; g__Gaetbulibacter | Control |
| T24 | ASV0003 | d__Bacteria; p__Proteobacteria; c__Gammaproteobacteria; o__Vibrionales; f__Vibrionaceae; g__Photobacterium; s__Photobacterium_damselae | Stress |
|  | ASV0048 | d__Bacteria; p__Bdellovibrionota; c__Bdellovibrionia; o__Bdellovibrionales; f__Bdellovibrionaceae; g__OM27_clade; s__uncultured_delta | Control |
|  | ASV0025 | d__Bacteria; p__Proteobacteria; c__Alphaproteobacteria; o__Rhodobacterales; f__Rhodobacteraceae | Control |
|  | ASV0027 | d__Bacteria; p__Verrucomicrobiota; c__Verrucomicrobiae; o__Verrucomicrobiales; f__Rubritaleaceae; g__Roseibacillus; s__uncultured_Verrucomicrobia | Control |
|  | ASV0022 | d__Bacteria; p__Bacteroidota; c__Bacteroidia; o__Cytophagales; f__Spirosomaceae; g__Taeseokella | Control |

**Table S3. LEfSe discovery of functional biomarkers in three levels of KEGG pathways.**

| **Time point** | **Ko No.** | **Level 1** | **Level 2** | **Level 3** | **Biomarkers (groups)** |
| --- | --- | --- | --- | --- | --- |
| All | ko05111 | Cellular Processes | Cellular community - prokaryotes | Vibrio cholerae pathogenic cycle | Stress |
|  | ko02040 |  | Cell motility | Flagellar assembly | Stress |
|  | ko02030 |  | Cell motility | Bacterial chemotaxis | Stress |
|  | ko04514 | Environmental Information Processing | Signaling molecules and interaction | Cell adhesion molecules (CAMs) | Control |
|  | ko00253 | Metabolism | Metabolism of terpenoids and polyketides | Tetracycline biosynthesis | Control |
|  | ko00625 |  | Xenobiotics biodegradation and metabolism | Chloroalkane and chloroalkene degradation | Control |
|  | ko00540 |  | Glycan biosynthesis and metabolism | Lipopolysaccharide biosynthesis | Stress |
|  | ko00121 |  | Lipid metabolism | Secondary bile acid biosynthesis | Stress |
| T06 | ko00625 | Metabolism | Xenobiotics biodegradation and metabolism | Chloroalkane and chloroalkene degradation | Control |
| T12 | ko05111 | Cellular Processes | Cellular community - prokaryotes | Vibrio cholerae pathogenic cycle | Stress |
|  | ko02040 |  | Cell motility | Flagellar assembly | Stress |
|  | ko02030 |  | Cell motility | Bacterial chemotaxis | Stress |
|  | ko00970 | Genetic Information Processing | Translation | Aminoacyl-tRNA biosynthesis | Control |
|  | ko03010 |  | Translation | Ribosome | Control |
|  | ko00253 | Metabolism | Metabolism of terpenoids and polyketides | Tetracycline biosynthesis | Control |
|  | ko01055 |  | Metabolism of terpenoids and polyketides | Biosynthesis of vancomycin group antibiotics | Control |
|  | ko00471 |  | Metabolism of other amino acids | D-Glutamine and D-glutamate metabolism | Control |
|  | ko00130 |  | Metabolism of cofactors and vitamins | Ubiquinone and other terpenoid-quinone biosynthesis | Stress |
|  | ko00121 |  | Lipid metabolism | Secondary bile acid biosynthesis | Stress |
| T24 | ko04112 | Cellular Processes | Cell growth and death | Cell cycle - Caulobacter | Control |
|  | ko00970 | Genetic Information Processing | Translation | Aminoacyl-tRNA biosynthesis | Control |
|  | ko03010 |  | Translation | Ribosome | Control |
|  | ko03430 |  | Replication and repair | Mismatch repair | Control |
|  | ko03440 |  | Replication and repair | Homologous recombination | Control |
|  | ko03020 |  | Translation | RNA polymerase | Control |
|  | ko01051 | Metabolism | Metabolism of terpenoids and polyketides | Biosynthesis of ansamycins | Control |
|  | ko00730 |  | Metabolism of cofactors and vitamins | Thiamine metabolism | Control |
|  | ko00471 |  | Metabolism of other amino acids | D-Glutamine and D-glutamate metabolism | Control |
|  | ko01055 |  | Metabolism of terpenoids and polyketides | Biosynthesis of vancomycin group antibiotics | Control |
|  | ko00860 |  | Metabolism of cofactors and vitamins | Porphyrin and chlorophyll metabolism | Control |
|  | ko00030 |  | Carbohydrate metabolism | Pentose phosphate pathway | Control |
|  | ko00900 |  | Metabolism of terpenoids and polyketides | Terpenoid backbone biosynthesis | Control |
|  | ko01040 |  | Lipid metabolism | Biosynthesis of unsaturated fatty acids | Stress |
|  | ko00650 |  | Carbohydrate metabolism | Butanoate metabolism | Stress |
|  | ko00072 |  | Lipid metabolism | Synthesis and degradation of ketone bodies | Stress |
|  | ko00020 |  | Carbohydrate metabolism | Citrate cycle (TCA cycle) | Stress |
|  | ko00511 |  | Glycan biosynthesis and metabolism | Other glycan degradation | Stress |
|  | ko00130 |  | Metabolism of cofactors and vitamins | Ubiquinone and other terpenoid-quinone biosynthesis | Stress |
|  | ko00281 |  | Metabolism of terpenoids and polyketides | Geraniol degradation | Stress |
|  | ko00480 |  | Metabolism of other amino acids | Glutathione metabolism | Stress |
|  | ko00785 |  | Metabolism of cofactors and vitamins | Lipoic acid metabolism | Stress |
|  | ko00540 |  | Glycan biosynthesis and metabolism | Lipopolysaccharide biosynthesis | Stress |
