# Supplementary material for: Low salinity stress increases the risk of Vibrio parahaemolyticus infection and gut microbiota dysbiosis in Pacific white shrimp
Source: BMC Microbiol. 2024 Jul 25;24:275. doi: 10.1186/s12866-024-03407-0 (PMC11271031; doi:10.1186/s12866-024-03407-0)
Supplement: Supplementary file 2 — Supplementary Material 2 [file 12866_2024_3407_MOESM2_ESM.docx]

**
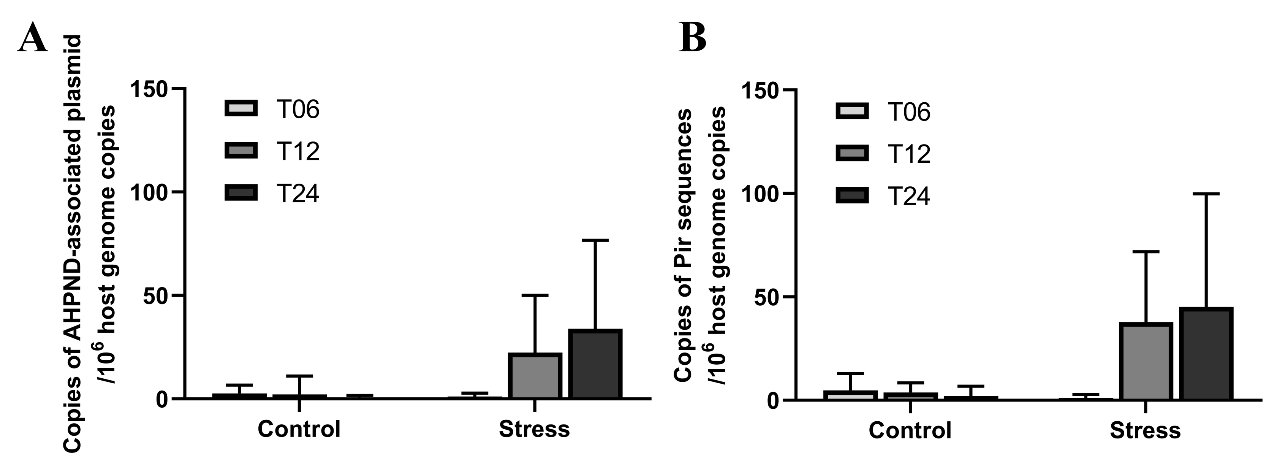
**

**Fig. S1 Time course of the relative copies of (A) the AHPND plasmid and (B) the toxin gene copies within Control and Stress groups.** The sample size was n=8 at each time point (T06, T12, and T24). The relative copies of the AHPND plasmid and toxin gene were normalized to the host genome copies.

**
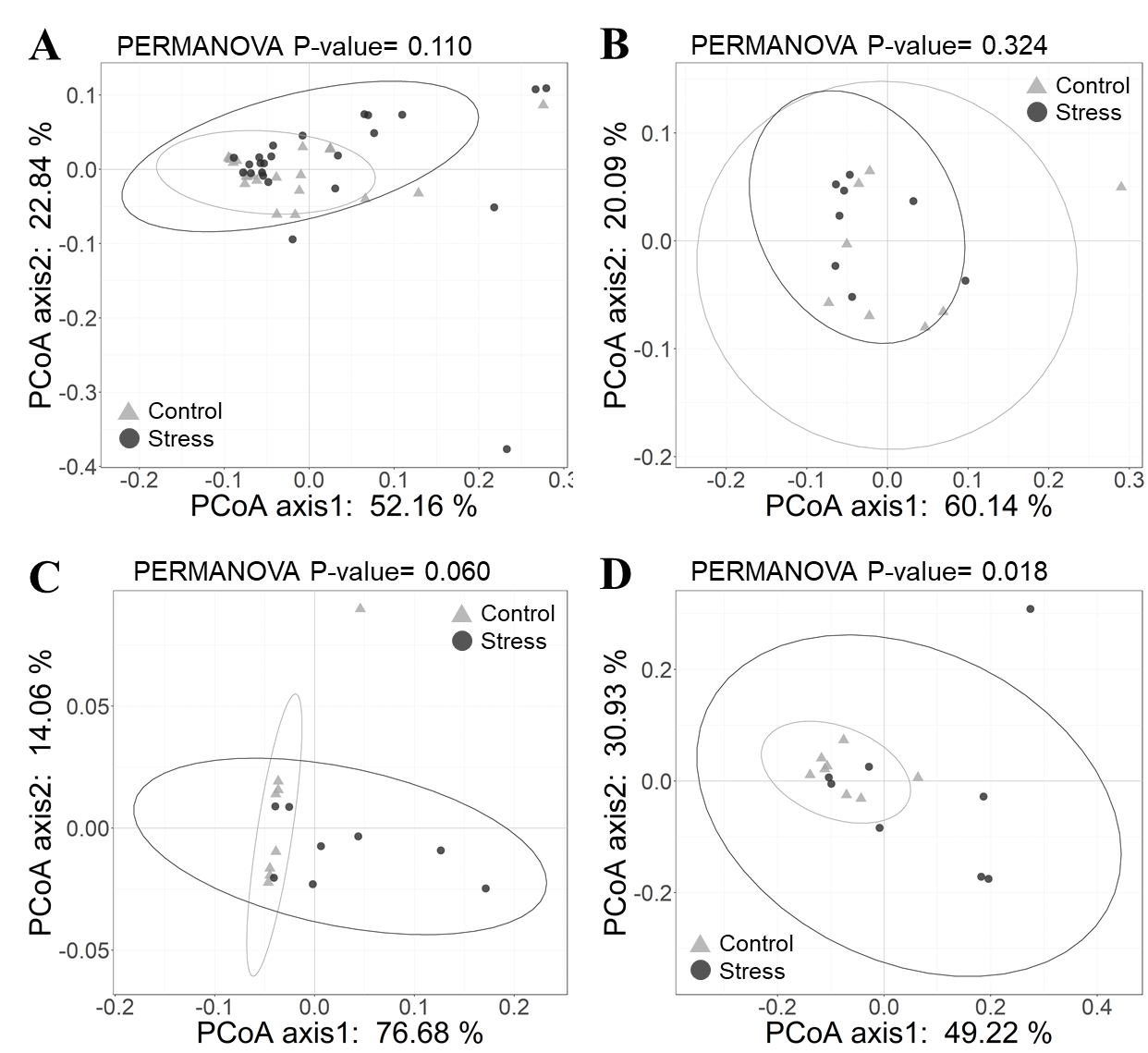
**

**Fig. S2 Principle coordinates analysis (PCoA) plots showing the dissimilarity of gut microbiota community composition based on the weighted UniFrac distances.** Each plot shows the pattern for (A) all time points, (B) T06, (C) T12, (D) T24 of the gut microbiota. Data points from Control and Stress experimental groups were labeled with different shapes and colors (Control=gray triangles, Stress=black dots). The sample size was n=8 at each time point. Statistical significance was calculated using the PERMANOVA test.


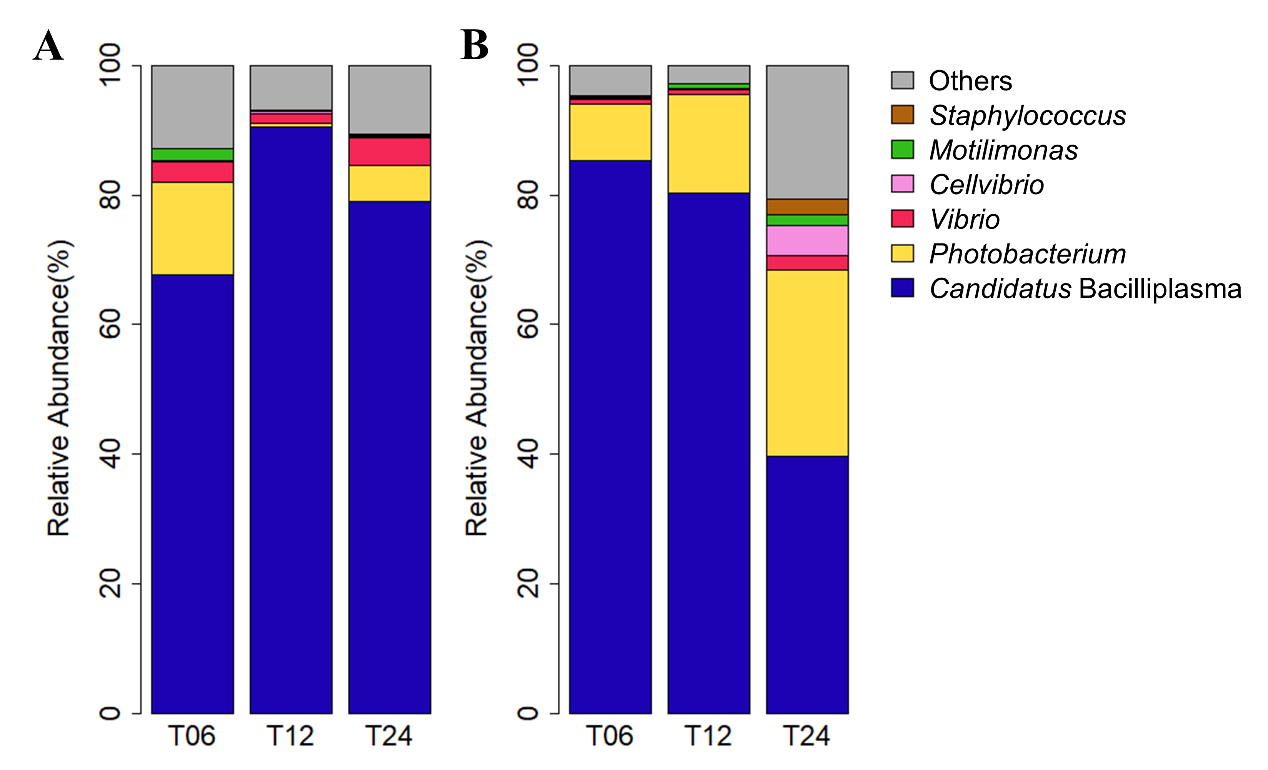


**Fig. S3 Bar plots showing differences in the taxonomic composition of shrimp gut microbiota between (A) Control and (B) Stress groups at each time point.** Six dominant bacterial genera (classified to the genus level with an average abundance greater than 0.5% across all samples) were shown in different colors, while the remaining genera were grouped as “Others” in gray. The sample size was n=8 at each time point (T06, T12, and T24).

**
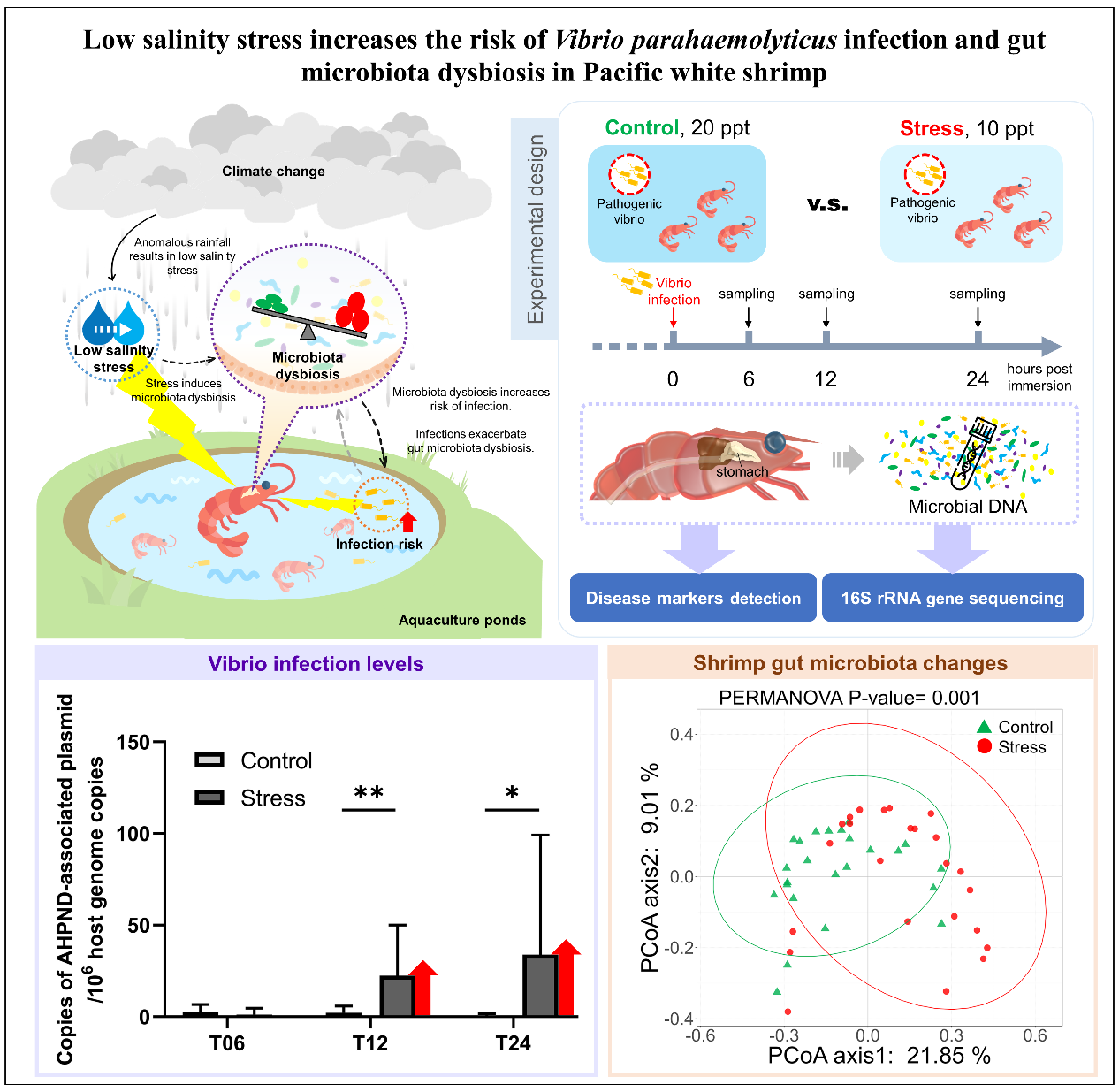
**

**Fig. S4 The graphical summary of this study.**
